# Spectrum and Frequency of Tumors, Cancer Risk and Survival in Chilean Families with Lynch Syndrome: Experience of the Implementation of a Registry

**DOI:** 10.3390/jcm9061861

**Published:** 2020-06-15

**Authors:** Karin Álvarez, Paulina Orellana, Marjorie De la Fuente, Tamara Canales, Eliana Pinto, Claudio Heine, Benjamín Solar, Claudia Hurtado, Pål Møller, Udo Kronberg, Alejandro José Zarate, Mev Dominguez-Valentin, Francisco López-Köstner

**Affiliations:** 1 Oncology and Molecular Genetic Laboratory, Coloproctology Unit, Clínica Las Condes, Santiago 7591047, Chile; kalvarez@clinicalascondes.cl (K.Á.); pau.orellana@gmail.com (P.O.); mdelafuentel@clinicalascondes.cl (M.D.l.F.); epinto@clinicalascondes.cl (E.P.); heinemed@gmail.com (C.H.); churtado@clinicalascondes.cl (C.H.); ukronberg@clinicalascondes.cl (U.K.); azarate@clinicalascondes.cl (A.J.Z.); 2 Cancer Institute, Clínica Las Condes, Santiago 7591047, Chile; tcanales@clinicalascondes.cl; 3 Colorectal Unit, Department of Surgery, Hospital San José, Osorno 5311523, Chile; 4 Genetic Section, University of Chile Clinic Hospital, Santiago 8380456, Chile; ben.solar.y@gmail.com; 5 Servicio de Neurología Infantil, Hospital de Puerto Montt, Puerto Montt 5507798, Chile; 6 Department of Tumor Biology, Institute for Cancer Research, The Norwegian Radium Hospital, 0369 Oslo, Norway; moller.pal@gmail.com (P.M.); mev.Dominguez.Valentin@rr-research.no (M.D.-V.); 7 Department of Surgery, Finis Terrae University, Santiago 7501015, Chile; 8 Instituto de Investigación, Universidad Católica de Trujillo, Chimbote 02800, Peru

**Keywords:** Lynch syndrome, mismatch repair gene, CRC, extracolonic tumors

## Abstract

Lynch syndrome (LS) is associated with the highest risk of colorectal (CRC) and several extracolonic cancers. In our effort to characterize LS families from Latin America, this study aimed to describe the spectrum of neoplasms and cancer risk by gender, age and gene, and survival in 34 Chilean LS families. Of them, 59% harbored *path_MLH1*, 23% *path_MSH2*, 12% *path_PMS2* and 6% *path_EPCAM* variants. A total of 866 individuals at risk were identified, of which 213 (24.6%) developed 308 neoplasms. In males, CRC was the most common cancer (72.6%), while females showed a greater frequency of extracolonic cancers (58.4%), including uterus and breast (*p* < 0.0001). The cumulative incidence of extracolonic cancers was higher in females than males (*p* = 0.001). *Path_MLH1* variants are significantly more associated with the development of CRC than extracolonic tumors (59.5% vs. 40.5%) when compared to *path_MSH2* (47.5% vs. 52.5%) variants (*p* = 0.05018). The cumulative incidence of CRC was higher in *path_MLH1*/*path_MSH2* carriers compared to *path_PMS2* carriers (*p* = 0.03). In addition, *path_MSH2* carriers showed higher risk of extracolonic tumors (*p* = 0.002). In conclusion, this study provides a snapshot of the LS profile from Chile and the current LS-associated diagnostic practice and output in Chile. Categorizing cancer risks associated with each population is relevant in the genetic counselling of LS patients.

## 1. Introduction

Colorectal cancer (CRC) is the third most common cause of cancer death in developed countries [1]. In Chile, DEIS-MINSAL (Department of Statistics and Health Information, Ministry of Health, 2016) reported 2362 deaths by CRC, being the third most common cause of cancer mortality followed by stomach cancer (3350 deaths) and trachea, bronchia and lung cancer (3162 deaths) [2]. During the last two decades, a progressive increase in mortality rates (116%) has been observed [3], probably due to risk factors related to increases in life expectancy, urban residence, lifestyle, nutrition and ethnic background. 

Heredity represents a major cause of CRC, with at least 20% of cases estimated to develop due to genetic factors and about 5% linked to inherited variants in cancer-predisposing genes [4,5,6,7]. Lynch syndrome (LS) is caused by a defective mismatch repair (MMR) system due to the presence of germline pathogenic variants in at least one of the MMR genes *MLH1*, *MSH2*, *MSH6* and *PMS2* or to deletions in the 3′ end of the *EPCAM* gene [8,9]. Such variants are here referred to as path_MMR, and, when specifying one of the genes, as *path_MLH1*, *path_MSH2*, *path_MSH6*, *path_PMS2* or *path_EPCAM*. *Path_MLH1* and *path_MSH2* variants are responsible for around 90% of the pathogenic variants identified in LS families while 7–10% are due to *path_MSH6* variants and less than 5% to *path_PMS2* variants. In addition, deletions in the *EPCAM* gene, which deactivate *MSH2*, account for approximately 1% of LS families [10,11]. 

Recent studies have reported that path_MMR carriers exhibit distinct patterns of cancer risk and survival according to affected gene, gender and age [11,12,13]. *Path_MLH1* and *path_MSH2* carriers showed similar high risk for CRC, endometrial and ovarian cancer, while *path_MSH2* carriers had higher risk for other cancers such as upper gastrointestinal, urinary tract, prostate and brain. On the other hand, *path_MSH6* carriers showed high endometrial cancer risk and a modestly increased CRC risk, and *path_PMS2* carriers showed a lower risk of any cancer [13]. Other tumor types, e.g., breast and pancreatic cancer, can come under discussion if represented as sporadic tumors within these families; they may indeed develop as part of the syndrome. This is suggested based on studies that showed lower MMR protein expression in extracolonic tumors from LS patients and their increased risk of developing these tumors in path_MMR carriers [14,15,16,17].

Several efforts have been made towards the genetic characterization of LS in Latin America [18,19,20,21], although there remains to be universal access to the genetic testing of all populations from our region or a better clinical characterization, which is relevant for the management and surveillance of high-risk patients and their families. Two studies from Brazil described the spectrum of tumors in LS families, showing that CRC was the most frequent, followed by extracolonic tumors, which were more frequent in women [22,23]. Some extracolonic tumors including breast and prostate have not been formally associated with LS [24,25]. In order to evaluate if the spectrum of CRC and extracolonic tumors may vary according to population, gene and gender, we aimed to describe the clinical and molecular characteristics of Chilean families with LS. 

## 2. Methods

### 2.1. Hereditary Colorectal Cancer Registry from Clinica Las Condes

A total of 125 Chilean families with a suspected clinical diagnosis of LS were recorded in our Hereditary Colorectal Cancer Registry in Clinica Las Condes (Santiago, Chile), which was started in 2003 (Figure S1). The registry includes families of patients with a diagnosis of CRC who were treated at our institution or who were referred by physicians from other health centers throughout Chile. 

LS families were enrolled according to the Amsterdam (AMS) criteria (I or II) [26,27], the Bethesda guidelines [28,29] and, since 2011, as a result of universal tumor screening for CRC [24]. The collected information included personal and family history, cancer diagnosis, age at onset, birth date, death date, pathology data and the results of DNA testing. All registered tumors were based on verbal information during interviews of the index cases. Clinical and pathological reports were recorded in cases where they were available. 

The study was conducted in accordance with the Declaration of Helsinki, and the protocol was approved by the Ethics Committee of Clinica Las Condes (project identification code O022018). All patients gave informed consent for their participation in the study.

Pedigree was constructed using the Progeny^®^ software (Progeny genetics, Delray Beach, FL, USA), including information from all relatives in at least the first and second degrees of maternal and paternal lineage. The available information on family history of cancer (colorectal and extracolonic) was documented for each proband, obtaining between 3 and 5 generations per family.

### 2.2. Genetic Analysis

Peripheral blood samples were collected into Ethylenediaminetetraacetic acid (EDTA) tubes, and genomic DNA was isolated using the MagNA Pure automated extraction system (Roche Diagnostics, Indeanapolis, IN, USA). In 2004–2014, the screening for pathogenic variants of *MLH1, MSH2, MSH6* and *PMS2* was performed using PCR amplification followed by single strand conformation polymorphisms (SSCP) and/or direct sequencing in a DNA automatic sequencer (Model ABI-3100, Applied Biosystems, Foster City, CA) as previously described [18]. For patients with no path_MMR identified, their samples were investigated for large genomic rearrangements involving *MLH1, MSH2, MSH6, PMS2* and *EPCAM* by multiplex ligation probe amplification (MLPA) using SALSA P003 and P072 kits according to the manufacturer’s specifications (MRC-Holland, Amsterdam, The Netherlands). Electropherograms were analyzed with the Coffalyser software. After 2015, newly enrolled families were studied by next generation sequencing (NGS) using a 5-cancer-associated genes panel (*MLH1, MSH2, MSH6, PMS2* and *EPCAM*) (Invitae, San Francisco, CA, USA). 

### 2.3. MMR Variant Nomenclature and Classification

The nomenclature guidelines of the Human Genome Variation Society (HGVS) [30] were used to describe the detected MMR variants. Variants were described by considering the following reference sequences: NM_000249.2 (*MLH1*), NM_000251.2 (*MSH2*) and NM_001322014.1 (*PMS2*). The MMR variants were classified, according to the International Society of Gastrointestinal Hereditary Tumors (InSIGHT), into the following categories: class 5 (pathogenic), class 4 (likely pathogenic), class 3 (uncertain), class 2 (likely not pathogenic) and class 1 (not pathogenic) [6].

The recurrence or novelty of the identified variants was established by interrogating four databases: the InSIGHT database (accessed via the Leiden Open Variation Database (LOVD)), the Universal Mutation Database (UMD), ClinVar and the Human Gene Mutation Database (HGMD), in March 2020.

### 2.4. Study Population

From the Hereditary Colorectal Cancer Registry, we selected all 34 LS families with an identified class 4 or 5 path_MMR variant. To characterize the type and frequency of neoplasms, we included family members (first- and second-degree relatives of the index case) who both had and had not performed genetic testing. The last revision of the information from the included families (including the review of death certificates in the national registry) was performed in March 2020. 

In order to analyze the LS families, we defined two groups:

Group 1 included all individuals at risk of cancer considering cases with a path_MMR variant, obligate carriers (who had 100% probability for being carriers due to their position in the pedigree in relation to relatives with proven mutation) and probable carriers by state (who had 50% probability of being carriers and had cancer). Because this group showed a high number of tumors, an initial characterization of the type and frequency of neoplasms was carried out. 

Group 2 included all proven mutation and obligate carriers with or without cancer who were under follow-up for their disease and family antecedents. In this group, we estimated the cumulative incidence of the first cancer (CRC and extracolonic) and the overall survival, defined as the time from diagnosis date until the date of death from any cause.

### 2.5. Statistical Analysis

The clinical characteristics were described using frequency distributions for categorical variables. A chi-square test was applied to determine the relationship between type of cancer and gender or path_MMR variants. All reported *p* values were two-sided, and statistical significance was set to the 95% level (*p*  <  0.05). 

The cumulative incidence of first CRC and extracolonic cancer diagnosed before 75 years of age was calculated using the Kaplan–Meier method and we compared the incidences curve using a log-rank test. Likewise, overall survival was estimated by the Kaplan–Meier method, and to minimize competitive causes of death, we restricted this calculation to cases diagnosed before 65 years of age. The results were obtained with a confidence interval of 95% and a *p* value <0.05 was considered statistically significant. The analyses were made using R Project version 3.5.3. 

Regarding ethical approval and consent to participate, all genetic tests were done with appropriate informed consent according to local requirements for healthcare and/or research.

## 3. Results

### 3.1. Genetic Characterization of LS Families

**Figure 1:**
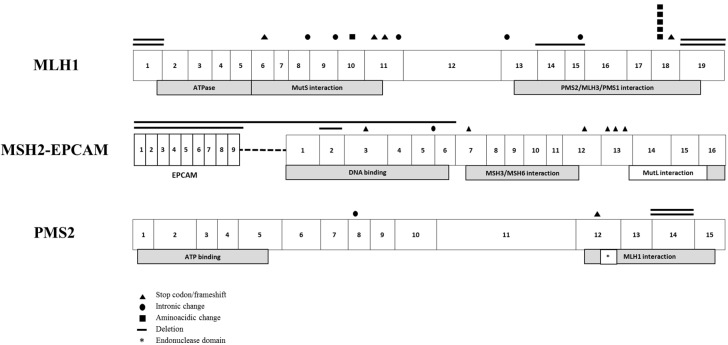
Schematic representation of the 27 different path_MMR variants identified in each gene.

From 125 families tested for MMR genes, we identified 34/125 (27%) carrying a class 4 or 5 path_MMR variant (Table 1). When the clinical criteria were considered, 27/34 (79%), 5/34 (15%) and 2/34 (6%) of the families fulfilled the Amsterdam criteria, Bethesda guidelines and universal screening criteria, respectively. More than half of the families harbored *path_MLH1* variants (*n* = 20, 59%), followed by eight families with *path_MSH2* (23%), four families with *path_PMS2* (12%) and two families with *path_EPCAM* (6%). For further analysis, *path_EPCAM* families were grouped with *path_MSH2* families. Notably, we did not identify *path_MSH6* variants in our cohort. Out of the 27 different pathogenic variants identified in these families, 11 (40.7%) were truncating, seven were intronic changes, seven large deletions and two non-synonymous amino acid changes. The schematic representation of path_MMR variants according to localization and type is summarized in Figure 1.

**Figure 2:**
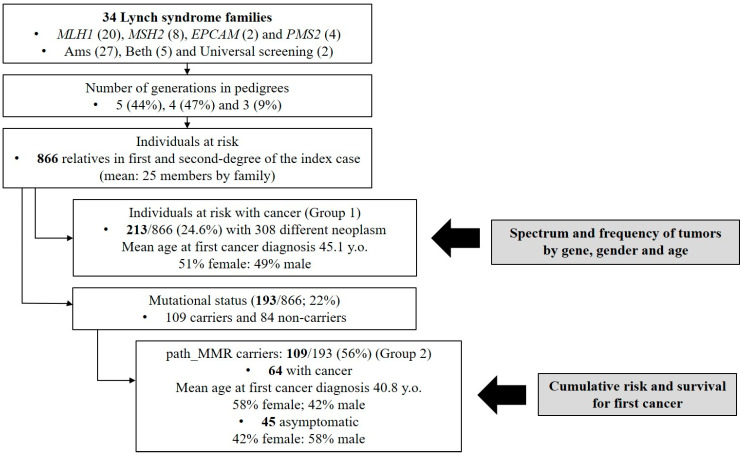
Flowchart of analysis.

Pedigree information from five, four and three generations was available in 44% (15/34), 47% (16/34) and 9% (3/34) of the families, respectively. Amongst all the individuals at risk, 866 relatives were identified with an average of 25 members per family (range 8–81). Of them, 213 individuals (24.6%) were diagnosed with 308 different neoplasms in total (mean 1.44 neoplasms per patient). These patients were defined as Group 1 and included path_MMR and obligate carriers as well as probable carriers by state. Fifty-one percent were female, and the mean age at any first cancer diagnosis was 45.1 years (Table 2). The workflow is summarized in Figure 2.

### 3.2. Clinicopathologic Features of LS Families

In Group 1, 308 different neoplasms were identified in 213 individuals. CRC was the most frequent tumor (170/308; 55%) identified in 140 individuals. The CRC anatomic distribution in the cecum (20.5%), appendix (1.3%), ascending colon (23.1%), hepatic flexure (6.4%), transverse colon (11.5%), splenic flexure (3.8%), descending colon (11.5%), sigmoid (11.5%), recto-sigmoid joint (2.6%) and rectum (7.7%) was identified. Thus, two thirds of tumors were diagnosed in the proximal colon. Moreover, 138/308 (45%) extracolonic tumors were identified in 111 subjects. Of these, 33, 20, 17, 13, and 13 had uterus, breast, stomach, urological (kidney/bladder/ureter) and skin cancer, respectively (Table 3).

As shown in Table S1, 57/213 patients developed synchronous and/or metachronous tumors. Thirty-six patients (16.9%) developed two neoplasms, while 4.1%, 3.6%, 1.4% and 0.5% developed three, four, five and six neoplasms, respectively. Most of the patients (14/57) developed colorectal synchronic and/or metachronous tumors, followed by colorectal and uterus (eight patients) and colorectal and skin (seven patients) cancers. It is important to mention that the time of diagnosis of metachronous neoplasms was performed predominantly between one and ten years after the first cancer diagnosis (31/74 neoplasms; 41%); however, some patients developed neoplasms up to 40 years after the first cancer.

### 3.3. Spectrum and Frequency of Tumors by Gene, Gender and Age

In Group 1, individuals at risk of cancer from families with *path_MLH1* variants mainly developed CRC (116/195; 59.5%) rather than extracolonic tumors (79/195; 40.5%), while individuals from families with *path_MSH2* showed CRC (47/99; 47.5%) and extracolonic tumors (52/99; 52.5%) equally (Table 3) (chi-square test *p* = 0.05018). In the case of *path_PMS2*, the frequency of CRC (7/14; 50%) and extracolonic tumors (7/14; 50%) was similar. Table 3 describes the type and frequency of the different extracolonic tumors according to genotype. In addition, extracolonic cancers without a previous history of CRC were identified in 35%, 52% and 45% of individuals from families with *path_MLH1*, *MSH2* and *PMS2* variants, respectively.

**Figure 3:**
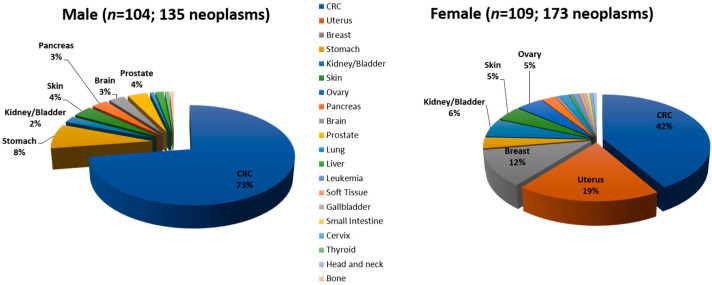
Spectrum and frequency of tumors by gender in Group 1.

When analyzing the frequency of tumors by gender in Group 1, 135 neoplasms in 104 men (1.29 neoplasms per man) and 173 neoplasms in 109 females (1.58 neoplasms per woman) were identified (Figure 3). In males, CRC was more frequent (98/135; 73.2%) than extracolonic tumors (37/135; 26.8%), and the most common extracolonic cancers included stomach (11 cases), skin (5 cases) and prostate (5 cases). On the other hand, extracolonic tumors (101/173; 57.6%) were more frequent than CRC (72/173; 42.4%) in women, including gynecological tumors (uterus and ovary with 33 and 8 cases, respectively), breast (20 cases) and urological (10 cases). A significant association between gender and type of cancer (CRC or extracolonic) was found (chi-square test *p* < 0.0001) (Table 2). Importantly, 84/213 (39%) individuals developed extracolonic cancer as the first tumor. 

Brain cancer was the earliest diagnosed neoplasm at mean 18 y (range 10–36). CRC and ovarian cancer were diagnosed at an average age of 44 years, followed by uterus, pancreas and stomach cancer, which were diagnosed at 48, 52 and 53 years, respectively. We also identified late onset tumors, including breast (mean 61 years), skin (64 years) and urological (66 years) cancer (Table S2). 

### 3.4. Characterization of Path_MMR Carriers

Due to Group 1’s inclusion of family members with unknown genetic status, we analyzed the frequency of tumors including only individuals with path_MMR variants (Group 2). Mutational status was defined in 193/866 relatives at risk through genetic analysis (188) and considering obligate carriers (5), among whom, 109 (56%) were carriers and 84 (44%) non-carriers of path_MMR variants. In Group 2, 45 (41%) were asymptomatic and 64 (59%) had developed cancer. Among the individuals with cancer, 123 different neoplasms were diagnosed (73 in females and 50 in males); the mean age at any first cancer diagnosis was 40.8 years old (Table 2; Figure 2). Sixteen patients died of LS at an average age of 58.5 years (range 39–87). At the time of writing, the current average age of living patients with cancer was 57 years (range 27–81) and of asymptomatic patients, 37.6 years (17–70). 

### 3.5. Path_ MMR Carriers: Tumor Spectrum, Cumulative Incidence and Survival Analysis

In the path_MMR carriers (Group 2), 90% of the tumors identified in men corresponded to CRC, while a lower frequency of CRC (58.9%) and a wide spectrum of extracolonic tumors (41.1%) was identified in females (chi-square test *p* = 0.00017), including gynecological (uterus (8%) and ovary (7%)), breast (8%) and skin (6%) cancers. CRC was the most frequent cancer (74% in *path_MLH1* carriers and 66% in *path_MSH2* carriers, chi-square test *p* = 0.3506) and extracolonic tumors were more recurrent in *path_MSH2* carriers (Table 2 and Table 3). These results are in line with those observed in Group 1.

**Figure 4:**
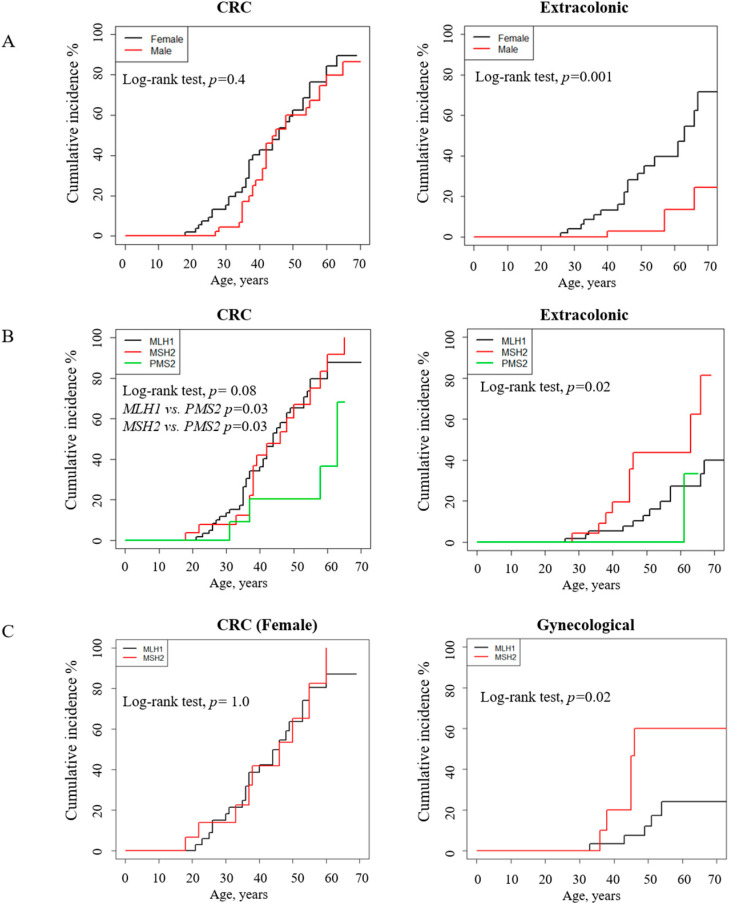
Cumulative incidence for first cancer diagnosed before 75 years in path_MMR carriers (Group 2) according to (**A**) gender, (**B**) gene and (**C**) females with *path_MLH1* and *path_MSH2*.

The cumulative incidence analysis of CRC and extracolonic first cancer before 75 years by gender or path_MMR was calculated via Kaplan–Meier analysis in Group 2 (Figure 4). Extracolonic cancer risk was significantly greater in females compared to males (log-rank test *p* = 0.001), with a cumulative risk of 31.3–71.7% vs. 2.7–24.3% at the age of 50–70, respectively. However, no significant difference in CRC risk according to gender was found (Figure 4A, Table 4).

*Path_MLH1* and *path_MSH2* carriers showed a higher cumulative CRC risk than *path_PMS2* carriers (Figure 4B, log-rank test *MLH1* vs. PMS2 *p* = 0.03; *MSH2* vs. *PMS2 p* = 0.03). The highest extracolonic cancer risk was found in *path_MSH2* carriers (log-rank test *p* = 0.02) (Figure 4B, Table 4). Due to uterus and ovary cancers being the most recurrent tumors in females, we analyzed the cumulative incidence of CRC and gynecological cancer according to genotype. Herein, we described that the risk of gynecological cancer was associated with *MSH2* (log-rank test *MLH1* vs. *MSH2 p* = 0.02) while no significant difference in CRC risk according to genotype was found (Figure 4C, Table 4).

Five- and ten-year crude survival analysis, focusing on the first CRC and extracolonic cancer diagnosed before 65 years of age, was performed. Eighty-nine percent of patients survived ten years or more after CRC diagnosis (95% CI 81.7–97.8) and 83.3% (95% CI 58.3–100) did so for uterus cancer.

## 4. Discussion

A more detailed characterization of gene- and gender-specific risk estimates in LS is crucial for updating clinical guidelines for the stratified surveillance, management and prevention of colorectal and extracolonic cancers [24,25]. Additionally, genetic background and environmental factors could explain the different phenotypes in different populations. Here, we present for the first time the tumor spectrum, incidence and survival, according to family history, clinical characteristics, gene and gender in families with LS from a Hereditary Colorectal Cancer Registry in Clinica Las Condes in Chile.

Our group has reported the profile of pathogenic variants in Chilean LS families since 2010 [18,30,31]. Genetic testing was initially performed for the *MLH1, MSH2, MSH6, EPCAM* and *PMS2* genes using SSCP, Sanger sequencing and MLPA analysis, in families that fulfilled the Amsterdam criteria (I or II) and the revised Bethesda guidelines. With the advent of next generation sequencing (NGS) technologies, we have included NGS panel testing and the implementation of universal tumor screening for CRC in our genetic testing practice. Currently, we have identified 27 pathogenic variants in MMR genes in 34 LS families. 

The distribution of pathogenic variants among the MMR genes in Chilean families showed a higher frequency in *path_MLH1* (59%), followed by *path_MSH2* (23%), *path_PMS2* (12%) and *path_EPCAM* (6%) variants. These results are in line with international databases and studies of Latin American LS families that have described a higher frequency of pathogenic variants in *MLH1* and *MSH2* genes [31,33]. Interestingly, in our cohort, no pathogenic variant was identified in *MSH6*, but on the other hand, we identified a higher frequency of *path_PMS2* variants and *EPCAM* deletions compared to those previously described [9,34]. This may be explained by the genetic differences between different populations or influences at the time of colonization, such as the Amerindian genetic component [18]. *Path_MSH6* variants are associated with a lower penetrance of CRC than *MLH1* and *MSH2* and an increased risk of endometrial cancer in females [35]; these characteristics could affect the identification of *path_MSH6* carrier families in our registry based on LS clinical criteria. For *path_PMS2* variants, we note that two out of four families were enrolled under universal screening and did not meet the classic Amsterdam or Bethesda criteria. This is consistent with the fact that variants in this gene are related to lower penetrance, cases of CRC at later age and higher risk of extracolonic cancer, decreasing the probability of meeting the classic clinical criteria.

In this study, we described how individuals at risk of cancer (Group 1) show a higher frequency of CRC in *path_MLH1* than in *path_MSH2* families (59% vs. 47%, *p* = 0.05018), along with a high risk of extracolonic cancer in *path_MSH2* carriers (Group 2), similarly to other studies [36,37,38,39]. In women, extracolonic tumors were more frequent than in men (58% vs. 27%, *p* < 0.0001). This association has been widely described in different populations centered in North America and Europe [40,41]. Gynecological and breast cancer were the most frequent extracolonic cancers in females from our LS cohort. Although uterine cancer (endometrial) has been widely described within the LS spectrum, breast cancer is still controversial. This is why some studies have described an increased risk of this cancer in path_MMR carriers [42,43]; in particular, *MLH1* carriers have been associated with a cumulative risk of 18.6% [44], *MSH6* with 31.1% and *PMS2* with 37.7% [42], while other studies found a marginally increased risk (12–15%) compared to the general population [35]. Efforts are ongoing to decipher the underlying tumorigenic processes involved in the development of breast cancer in patients with Lynch syndrome [45,46]. However, sporadic cases of breast cancer cannot be ruled out in our cohort due to the late age at diagnosis and the high frequency in the Chilean general population, where breast cancer was the main cause of mortality by malignant tumors reaching a rate of 15.5:100,000 for women in Chile [2].

In the present study, gastric cancer was the third most frequent extracolonic tumor in our cohort, and the first one in men. Considering that gastric cancer is the most frequent cause of mortality by malignant tumors in both genders in Chile [2] and is related to *Helicobacter pylori* infection [47], the influence of this environmental factor could modify the risk of gastric cancer in path_MMR carriers. Similar studies also observed a high frequency of gastric cancer in LS patients associated with *Helicobacter pylori* infection [48], suggesting endoscopic surveillance and bacterial eradication in these patients according to the guidelines [24,25].

Other extracolonic tumors identified in our LS families, such as pancreatic, prostate, skin and urological cancer, have also been described in other LS populations, with frequencies and risks similar to those described here [49,50,51,52,53,54]. 

The implementation of a hereditary cancer registry allows the identification of individuals at risk, genetic testing, genetic-clinical counseling, early detection of cancer, education of families, and training of professionals, thereby reducing the mortality and morbidity of LS patients [55]. In this sense, the favorable survival observed in our cohort might be explained either by a better prognosis in LS patients [56,57] or by the importance of a Hereditary Registry where a protocolized follow-up was previously defined for our institution and shared with other referral physicians in contact with our registry. Our registry was one of the first to be implemented in South America and constitutes a “reference clinic for genetic counselling” in Chile. As in most of the countries in our region, there is still a lack of awareness about hereditary cancer; we have initiated an e-learning course in cancer genetic counselling with the aim of providing a specialized training course in cancer genetic counselling to Latin American healthcare professionals. Additionally, we are investigating the genetic and clinical differences between populations in Latin America as we are part of a network of Latin American LS family registries (LA-GETH) [33,58]. This study contributes to the clinical and genetic description of Chilean LS families over the last 17 years. 

## 5. Conclusions

In conclusion, this study provides a snapshot view of the current LS-associated diagnostic practice/output in Chile. Categorizing cancer risk according to age, gender, path_MMR and other factors associated with each population (genetic and environmental) is relevant in the genetic counseling of LS patients, facilitating an early diagnosis of associated cancers and improving disease prognosis.

## Figures and Tables

**Table 1:** Summary of class 4 and 5 path_MMR variants identified in 34 Chilean LS families.

Family	Criteria #	Gene	Nucleotide Change ##	Protein Change
HNPCC056	Ams	*MLH1*	Deletion exon 1 ^b^	p.0?
HNPCC008	Ams	*MLH1*	Deletion exon 1 ^b^	*p*.0?
HNPCC006	Ams	*MLH1*	c.503dupA ^a^	p.N168Kfs*4
HNPCC002	Ams	*MLH1*	c.677 + 5G > A ^b^	p.Q197Rfs*8/p.E53Ffs*8
HNPCC029	Ams	*MLH1*	c.790 + 1G > A ^a^	p.E227_S295del
HNPCC043	Ams	*MLH1*	c.794G > C ^c^	p.R265P
HNPCC009	Ams	*MLH1*	c.901C > T ^a^	p.Q301*
HNPCC080	Ams	*MLH1*	c.997_1000delAAGC ^c^	p.K333Sfs*33
HNPCC037	Ams	*MLH1*	c.1038 + 1G > T ^b^	p.T347Ffs*14
HNPCC062	Ams	*MLH1*	c.1559 − 2A > C ^c^	p.L521Kfs*34
HNPCC018	Ams	*MLH1*	Deletion exons 14 and 15 ^b^	p.V520Gfs*7
HNPCC001	Ams	*MLH1*	c.1731 + 3A > T ^a^	p.S556Rfs*14
HNPCC073	Ams	*MLH1*	c.2041G > A ^a^	p.A681T
HNPCC082	Ams	*MLH1*	c.2041G > A ^a^	p.A681T
HNPCC086	Beth	*MLH1*	c.2041G > A ^a^	p.A681T
HNPCC011	Beth	*MLH1*	c.2041G > A ^a^	p.A681T
HNPCC057	Ams	*MLH1*	c.2041G > A ^a^	p.A681T
HNPCC019	Beth	*MLH1*	c.2092_2093delTC ^a^	p.S698Rfs*5
HNPCC004	Ams	*MLH1*	Deletion exon 19 ^b^	p.S702_X757del
HNPCC010	Ams	*MLH1*	Deletion exon 19 ^b^	p.S702_X757del
HNPCC047	Ams	*MSH2*	Deletion exon 2 ^b^	p.A72Ffs*9
HNPCC100	Ams	*MSH2*	c.388_389delCA ^c^	p.Q130Vfs*2
HNPCC118	Ams	*MSH2*	c.942 + 3A > T ^c^	p.V265_Q314del
HNPCC027	Ams	*MSH2*	c.1215C > A ^a^	p.Y405*
HNPCC111	Beth	*MSH2*	c.1861C > T ^c^	p.R621*
HNPCC031	Ams	*MSH2*	c.2038C > T ^a^	p.R680*
HNPCC075	Ams	*MSH2*	c.2131C > T ^c^	p.R711*
HNPCC012	Ams	*MSH2*	c.2185_2192del7insCCCT ^a^	p.M729_E731delinsP729_*730
HNPCC088	Ams	*EPCAM-MSH2*	Deletion *EPCAM* and exons 1–6 *MSH2* ^c^	p.?
HNPCC094	Ams	*EPCAM*	Deletion exons 6–9 ^c^	p.?
HNPCC106	US	*PMS2*	c.903G > T ^d^	p.K301N (Skips exon 8)
HNPCC084	Beth	*PMS2*	c.2016delG ^c^	p.M672Ifs*15
HNPCC093	Ams	*PMS2*	Deletion exon 14 ^c^	p.A759Gfs*8
HNPCC116	US	*PMS2*	Deletion exon 14 ^c^	p.A759Gfs*8

# Ams: Amsterdam; Beth: Bethesda; US: Universal Screening; ## path_MMR variants have been described in ^a^ Alvarez et al. 2010 [18]; ^b^ Wielandt et al. 2012 [30]; ^c^ Rossi et al. 2017 [31], ^d^ Senter et al. 2008 [32].

**Table 2:** Clinical description of individuals with cancer in Group 1 and Group 2.

Clinical Information	Group 1	Group 2
Gender: *n* (%)		
Female	109 (51)	37 (59)
Male	104 (49)	27 (42)
Total	213	64
Neoplasms: *n* (%)		
CRC	170/140 subjects	88/61 subjects
Extracolonic cancer	138/109 subjects	35/23 subjects
Total	308	123
Neoplasm by gender **: *n* (%)		
Female	173	73
CRC	72 (41.6)	43 (58.9)
Extracolonic cancer	101 (58.4)	30 (41.1)
Male	135	50
CRC	98 (72.6)	45 (90)
Extracolonic cancer	37 (27.4)	5 (10)
Age (years) at diagnosis: mean and range		
Any first cancer	45.1 (1–89)	40.8 (18–84)
Female	45.9 (1–89)	39.5 (18–84)
Male	44.2 (10–84)	42.5 (27–65)
First CRC	43.4 (18–89)	41.2 (18–65)
Female	43.1 (18–89)	39.6 (18–63)
Male	42.5 (21–73)	43.2 (27–65)
First extracolonic cancer	51.0 (1–88)	43.3 (26–84)
Female	50.8 (1–88)	48.1 (26–84)
Male	51.5 (10–84)	55.0 (40–66)

CRC: colorectal cancer; ** Chi-square test between gender and type of cancer (CRC and extracolonic) showed *p* < 0.0001 (Group 1) and *p* = 0.00017 (Group 2).

**Table 3:** Spectrum and frequency of neoplasms in Group 1 and Group 2 for each path_MMR gene.

	Group 1				Group 2			
Neoplasm	*path_MLH1* *n* = 195	*path_MSH2* *n* = 99	*path_PMS2* *n* = 14	Total *n* = 308	*path_MLH1* *n* = 77	*path_MSH2* *n* = 41	*path_PMS2* *n* = 5	Total *n* = 123
Colorectal: *n* (%)	116 (59.5)	47 (47.5)	7 (50)	170	57 (74)	27 (66)	4 (80)	88
Extracolonic: *n* (%)	79 (40.5)	52 (52.5)	7 (50)	138	20 (26)	14 (34)	1 (20)	35
Gynecological cancer								
Uterus	21 (10.8)	11 (11.1)	1 (7.1)	33	4 (5.2)	2 (4.9)	0	6
Ovary	2 (1.0)	5 (5.1)	1 (7.1)	8	1 (1.3)	4 (9.8)	0	5
Upper gastrointestinal cancer								
Stomach	10 (5.1)	7 (7.1)	0	17	1 (1.3)	0	0	1
Pancreas	6 (3.0)	1 (1.0)	1 (7.1)	8	2 (2.6)	0	0	2
Liver	3 (1.5)	0	0	3	0	0	0	0
Gallbladder	1 (0.5)	0	0	1	0	0	0	0
Small Intestine	0	1 (1.0)	0	1	0	1 (2.4)	0	1
Genitourinary tract cancer								
Kidney	2 (1.0)	6 (6.1)	1 (7.1)	9	1 (1.3)	1 (2.4))	0	2
Bladder	0	1(1.0)	0	1	0	1(2.4)	0	1
Ureter	3(1.5)	0	0	3	0	0	0	0
Other cancers								
Breast	12 (6.2)	7 (7.1)	1 (7.1)	20	4 (5.2)	2 (4.9)	0	6
Skin	7 (3.6)	6 (6.1)	0	13	5 (6.5)	3 (7.3)	0	8
Brain	2 (1.0)	3 (3.0)	0	5	0	0	0	0
Lung	2 (1.0)	2 (2.0)	0	4	1 (1.3)	0	0	1
Prostate	5 (2.6)	0	0	5	0	0	0	0
Soft tissue	1 (0.5)	0	0	1	1(1.3)	0	0	1
Bone	1 (0.5)	0	0	1	0	0	0	0
Leukemia	1 (0.5)	0	1 (7.1)	2	0	0	0	0
Head and Neck	0	1 (1.0)	0	1	0	0	0	0
Thyroid	0	1 (1.0)	0	1	0	0	0	0
Cervix	0	0	1 (7.1)	1	0	0	1 (20)	1

A chi-square test between *path_MLH1/MSH2* and type of cancer (CRC and extracolonic) showed *p* = 0.05018 (Group 1) and *p* = 0.3506 (Group 2).

**Table 4:** Cumulative incidence (%) of colorectal and extracolonic cancers diagnosed before 75 years by age, gender and gene in path_MMR carriers (Group 2).

Cancer Type	Age, Year	All %	CI95	Gender				*path_MLH1*				*path_MSH2*				*path_PMS2* **	
				Female	CI95	Male	CI95	Female	CI95	Male	CI95	Female	CI95	Male	CI95	Both	CI95
Colorectal (*n* = 61)	50	59.5	46.4–69.4	59.5	49.1–72.2	60.1	38.9–74.0	63.5	39.1–78.2	67.9	39.7–83.0	53.5	9.8–76.0	68.0	9.2–88.7	20.5	0–42.3
	60	75.7	62.3–84.4	76.5	56.2–87.4	74.6	52.9–86.3	80.5	52.1–92.0	78.6	50.1–90.8	82.6	10.4–96.6	84.0	9.3–97.2	36.4	0–63.1
	70	88.2	73.7–94.7	89.5	66.6–96.7	86.5	59.3–95.5	87.0	56.7–96.1	89.3	45.7–97.9	100.0	-	100.0	-	68.2	0–92.8
Extracolonic (*n* = 22)	50	18.5	8.6–27.3	31.3	14.4–44.9	2.7	0–7.8	23.2	4.6–38.1	0	-	64.1	12.8–85.2	10.0	0–26.8	0.0	-
	60	28.0	14.8–39.2	39.6	19.7–54.5	13.5	0–27.2	36.6	10.2–55.2	15.4	0–32.9	64.1	12.8–85.2	10.0	0–26.8	0.0	-
	70	50.0	27.9–65.4	71.7	35.0–87.7	24.3	0–44.7	60.4	15.7–81.4	15.4	0–32.9	100	-	55.0	0–88.9	33.3	0–70.0
Gynecological (*n* = 10)	50	22.1	7.1–34.7	22.1	7.1–34.7	-	-	12.1	0–24.2	-	-	60.0	5.7–83.0	-	-	0.0	-
	60	30.0	11.8–44.4	30.0	11.8–44.4	-	-	24.2	2.1–41.2	-	-	60.0	5.7–83.0	-	-	0.0	-
	70	30.0	11.8–44.4	30.0	11.8–44.4	-	-	24.2	2.1–41.2	-	-	60.0	5.7–83.0	-	-	0.0	-
Skin (*n* = 5)	50	2.4	0–5.6	2.1	0–6.0	2.7	0–7.8	0.0	-	0.0	-	7.7	0–21.1	10.0	0–26.8	0.0	-
	60	5.0	0–10.7	2.1	0–6.0	8.1	0–18.8	0.0	-	7.7	0–21.1	7.7	0–21.1	10.0	0–26.8	0.0	-
	70	14.5	0–27.0	11.0	0–26.5	18.3	0–37.2	12.5	0–32.7	7.7	0–21.1	7.7	0–21.1	55.0	0–88.9	0.0	-
Upper gastrointestinal tract (*n* = 3)	50	1.7	0–5.0	-	-	-	-	-	-	-	-	-	-	-	-	-	-
	60	4.3	0–10.1	-	-	-	-	-	-	-	-	-	-	-	-	-	-
	70	4.3	0–10.1	-	-	-	-	-	-	-	-	-	-	-	-	-	-
Breast (*n* = 3)	50	5.2	0–12.1	5.2	0–12.1	-	-	3.4	0–9.9	-	-	12.5	0–32.7	-	-	0.0	-
	60	9.0	0–18.5	9.0	0–18.5	-	-	9.5	0–21.6	-	-	12.5	0–32.7	-	-	0.0	-
	70	9.0	0–18.5	9.0	0–18.5	-	-	9.5	0–21.6	-		12.5	0–32.7	-	-	0.0	-

** For PMS2 both genders were evaluated.

## Supplementary Materials

The following are available online at https://www.mdpi.com/2077-0383/9/6/1861/s1, Figure S1: Flowchart of Chilean families from Hereditary Colorectal Cancer Registry in Clínica Las Condes; Table S1: Summary of synchronous and metachronous neoplasms in 57 patients from Group 1; Table S2: Summary of mean age at diagnosis and range of major neoplasms observed in LS Chilean families.

## Abbreviations

AMS	Amsterdam
CRC	colorectal cancer
HGMD	Human Gene Mutation Database
HGVS	Human Genome Variation Society
InSIGHT	International Society of Gastrointestinal Hereditary Tumors
LS	Lynch syndrome
LOVD	Leiden Open Variation Database
MLPA	Multiplex ligation dependent-probes amplification
MMR	mismatch-repair gene
MSI	microsatellite instability
NGS	Next generation sequencing
Path_MMR	Pathogenic (disease-causing) variant of an MMR gene.
*path_MLH1*	Pathogenic (disease-causing) variant of the *MLH1* gene
*path_MSH2*	Pathogenic (disease-causing) variant of the *MSH2* gene
*path_MSH6*	Pathogenic (disease-causing) variant of the *MSH6* gene
*path_PMS2*	Pathogenic (disease-causing) variant of the *PMS2* gene
*path_EPCAM*	Pathogenic (disease-causing) variant of the *EPCAM* gene
UMD	Universal Mutation Database
