# Supplementary material for: Spectrum and Frequency of Tumors, Cancer Risk and Survival in Chilean Families with Lynch Syndrome: Experience of the Implementation of a Registry
Source: J Clin Med. 2020 Jun 15;9(6):1861. doi: 10.3390/jcm9061861 (PMC7356331; doi:10.3390/jcm9061861)
Supplement: Supplementary file 1 [file jcm-09-01861-s001.pdf]

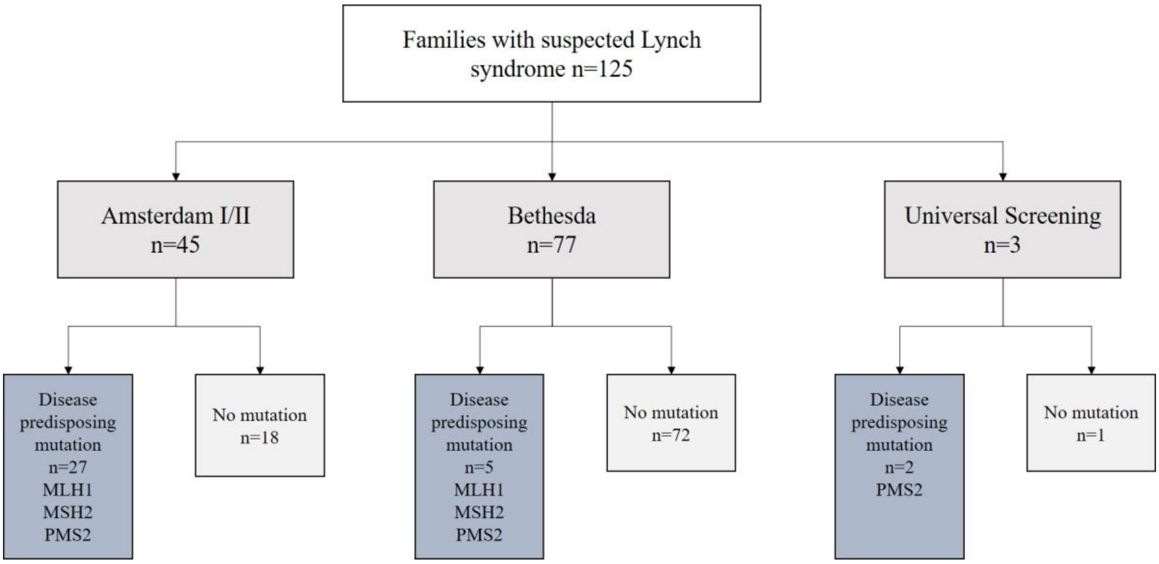

Figure S1. Flowchart of Chilean families from Hereditary Colorectal Cancer Registry in Clínica Las Condes.

Table S1. Summary of synchronous and metachronous neoplasms in 57 patients from Group 1.

**Synchronous and metachronous neoplasms**	**Number of patients (%)**
Two	36 (16.9)
Three	9 (4.1)
Four	8 (3.6)
Five	3 (1.4)
Six	1 (0.5)
Localization	Number of patients
Colorectal (2–5 tumors)	14
Colorectal and uterus	8
Colorectal and skin	7
Colorectal and ovary	3
Colorectal and stomach	3
Colorectal, breast and uterus	3
Colorectal and breast	1
Colorectal and kidney/ureter/bladder	2
Colorectal, kidney/bladder and small bowel	1
Colorectal and cervix	1
Colorectal and pancreas	1
Colorectal, pancreas and breast	1
Colorectal, pancreas and skin	1
Colorectal and prostate	1
Colorectal and soft tissue	1
Colorectal, uterus, ovary and kidney	1
Stomach (2 tumors)	1
Uterus and ovary	1
Uterus and kidney	1
Uterus and breast	1
Uterus and leukemia	1
Uterus and gallbladder	1
Breast, skin, kidney/bladder and head and neck	1
Time of metachronous cancer diagnosis since the first neoplasm (years)	*n*
1–10	31
11–20	18
21–30	20
31–40	4
41–50	1

Table S2. Summary of mean age at diagnosis and range of major neoplasms observed in LS Chilean families (Group 1).

**Neoplasm**	***n***	**Mean age at diagnosis (years)**	**Range (years)**
Brain	5	18.0	10–36
CRC	133	43.4	18–89
Ovary	7	44.9	36–54
Uterus	26	48.5	25–67
Pancreas	6	52.2	17–77
Stomach	13	53.7	32–70
Breast	13	58.5	32–84
Skin	9	63.8	28–88
Kidney/ureter/bladder	10	64.9	52–78
